# Role of community pharmacists in cardiovascular diseases-related health promotion and dyslipidemia management in Malaysia: A nationwide cross-sectional study

**DOI:** 10.1371/journal.pone.0290883

**Published:** 2023-09-28

**Authors:** Farhana Fakhira Ismail, Adyani Md Redzuan, Wei Wen Chong, Mariani Ahmad Nizaruddin

**Affiliations:** 1 Centre for Quality Management of Medicine, Faculty of Pharmacy, Universiti Kebangsaan Malaysia, Kuala Lumpur, Malaysia; 2 Department of Clinical Pharmacy, Faculty of Pharmacy, Universiti Teknologi MARA, Selangor, Malaysia; 3 Department of Community and Pharmacy Practice, Faculty of Pharmacy, University of Cyberjaya, Cyberjaya, Selangor, Malaysia; Lahore Medical and Dental College, PAKISTAN

## Abstract

**Background:**

Cardiovascular disease (CVD) is a leading cause of death and disability worldwide, imposing a significant burden on patients and healthcare systems. The role of pharmacists in reducing cardiovascular disease (CVD) is pivotal as they play an essential part in the healthcare team, particularly in medication management and patient education. Pharmacists are well-positioned to contribute to the prevention and control of CVD through various roles, including medication management and patient education. This study aims to investigate the current involvement of community pharmacists in Malaysia, specifically in cardiovascular diseases-related health promotion activities and dyslipidemia management, including their perceived barriers.

**Method:**

This cross-sectional survey was conducted among community pharmacists in all 14 states of Malaysia between November 2021 and July 2022. The self-administered survey was shared to relevant groups through various social media platforms.

**Results:**

A total of 312 community pharmacists were involved in the survey. Majority of the respondents were females (66%), with a mean age (SD) of 32.9 (8.4) years. Most of the respondents showed satisfactory practice for patient counselling, but improvements are needed particularly in risk assessment and collaborative care aspect. Most of them expressed their interest for dyslipidemia management training (89.4%). Lack of access to medical records (71.2%) and lack of CVD-related educational materials (70.8%) were the two main perceived barriers identified.

**Conclusion:**

Community pharmacists in Malaysia provide a satisfactory role in the provision of cardiovascular disease-related health promotion activities, especially in providing patient counselling. Strengthening collaborative care is essential for providing comprehensive and patient-centered intervention in dyslipidemia management. This requires ongoing efforts to address and overcome existing barriers for effective teamwork and coordination among healthcare professionals.

## Introduction

According to Global Burden of Diseases, studies showed that low-density lipoprotein cholesterol (LDL-C) accounted for 4.4 million deaths around the world in 2019 [1]. In Europe, cardiovascular disease (CVD) is the primary cause of death, illness, and decreased quality of life, and is connected to a heavy clinical and financial burden [2–4]. CVD accounts for 27% of all deaths and is equivalent to one fatality every three minutes [2–4]. In Asia, the number of CVD mortalities has nearly doubled over the past 30 years, from 5.6 million to 10.8 million [5]. Dyslipidemia is one of the highly prevalent risk factors for CVDs among Malaysians [6] with 64% of Malaysian adults had elevated total cholesterol (TC), and 56.7% had elevated low-density lipoprotein cholesterol (LDL-C) [6].

A prospective study conducted in Malaysia from 2011 to 2016 involving 11,288 respondents revealed that 74.2% of the respondents with CVD were not on lipid-lowering medicines [7]. The cornerstone of managing dyslipidemia is the combination of therapeutic lifestyle changes and lipid-lowering medicines [8–11]. According to national and international guidelines, those at a high risk of developing cardiovascular disease (CVD) should be prescribed lipid-lowering medicines, particularly statins, for both primary and secondary prevention. Consistently, all of these recommendations urge stratifying patients’ CVD risks in order to direct lipid-lowering medicines use and therapeutic lifestyle changes to establish each patient’s treatment goals [8–11].

Pharmacists in the community are well-positioned to assist patients with established CVD or who are at risk of developing this [12]. In many industrialised and developing nations, community pharmacists (CPs) are the first point of contact in the healthcare system due to their ease of access and perceived affordability [13]. Studies have shown that controlling dyslipidemia, hypertension, and secondary preventive medicines can improve health outcomes for patients with CVD in terms of improvement in lipid parameters and reduction in CVD risk score. In addition, risky behaviours and risk factors for CVD have been reduced as a result of community pharmacy-based interventions [14, 15].

Recent study by Hwang and Smith (2019) emphasized that major guidelines have urged for engagement between pharmacists and the society [16]. However, additional research is warranted to study the execution method so that the engagement would be optimize and beneficial. The authors further mentioned that action is needed to inform the best way to engage with the CPs who remain as untapped yet very powerful resources in CVD prevention and dyslipidemia management. This engagement would provide better health outcomes for the society [16]. Therefore, the purpose of this study is to investigate the current involvement of CPs in Malaysia, specifically in dyslipidemia management, including their perceived barriers in providing CVD and dyslipidemia services.

## Methods

The study received ethics approval from the Research Ethics Committee of Universiti Kebangsaan Malaysia (UKM PPI/111/8/JEP-2021-513).

### Study design and participants

This cross-sectional survey was conducted among CPs in all 14 states of Malaysia between November 2021 to July 2022. Using Raosoft calculation, the target sample size is 343, with a ±5% margin of error and a confidence level of 95% [17]. Calculated sample size is based on the latest number of registered community pharmacists in the country which is 3078. This information was obtained from website of Pharmacy Division, Ministry of Health, Malaysia [18]. The self-administered survey was conducted online via Google Form and shared in relevant groups involving CPs on Facebook, Whatsapp and Telegram platforms. Several strategies were used to reach as many respondents as possible from all states of Malaysia and from all types of pharmacy, either chain or independent pharmacy. Professional and personal networks of the researchers was used to reach the pharmacy’s association leaders to disseminate the survey. Only the first and corresponding authors have access to the information of the participants. The inclusion criteria of the study include CPs practicing in Malaysia either as full-time, part-time or locum, registered with the Pharmacy Board of Malaysia and owned a License A. License A holder is a license given to the registered pharmacist to import, store and manage all medications by retail or retail and wholesale. Written consent was obtained at the initial part of the questionnaire, prior to answering the first section of the questionnaire. CPs who were unable to grant consent to participate in the study or who were training pharmacists (known as Provisionally Registered Pharmacists (PRP) throughout the study were excluded.

### Study instrument

The questionnaire was adapted from El Hajj and colleagues who had used the questionnaire and conducted the study in Qatar [19]. Permission had been granted for the questionnaire to be used in this study. For face and content validity, the first version of the adapted questionnaire was reviewed by two academicians withestablished background in CVD research in community pharmacy, and two community pharmacists. The content validity and clarity of the questions were reviewed. The questionnaire was then pilot tested among 20 community pharmacists for further improvement. The Cronbach alpha determined for each subscale was as follows CVD involvement subscale (Cronbach alpha = 0.851), dyslipidemia involvement subscale (Cronbach alpha = 0.92), perceived barriers subscale (Cronbach alpha = 0.548), and finally contents of educational materials subscale (Cronbach alpha = 0.792)Other than measuring the Cronbach–alpha, the participants from the pilot study were requested to provide feedback on the questionnaire’s face and content validity, the appropriateness of the response alternatives, whether any items needed to be updated, removed, or added, and how the survey’s layout could be improved. Questions relevance, necessity and clarity were reviewed thoroughly. Modifications were made based on the feedback given. The questionnaire was then distributed using Google Form by convenience sampling method.

The final structured questionnaire consisted of 5 separate sections. Only respondents who consented to participate in this study were involved. Section 1 is the sociodemographic data of the respondents. Relevant data was asked in this section especially pertaining to the pharmacy’s information and the CP’s background. Section 2 was aimed to determine the current involvement of CPs in CVD-related health promotion activities. The CPs were asked to rate their current involvement using a Likert scale (Never, Rarely, Often, Always). In addition to that, in section 3, the CPs were asked on how often they offer dyslipidemia care services. In order to determine the factors influencing the CVD health promotional activities with dyslipidemia care services, for section 2 and 3, a scoring method was done where the participants will be scored with ‘0’ for ‘never’, ‘1’ for ‘rarely’, ‘2’ for ‘often’ and ‘3’ for always. The total score will be added including all statements/questions for that section. The perceived barriers were also identified in section 4. This is done by listing all possible barriers that may be encountered by the CPs and they may choose more than one option. Finally, in section 5, CPs were asked to choose the contents of educational materials which they think appropriate to be included.

### Data analysis

The statistical analysis in this study was done using IBM® Statistical Package for Social Science (SPSS) version 28. Descriptive statistics was used where the socio-demographic and practice characteristics were summarized using frequency distribution except for age where the mean and standard deviation were used. The score of CVD related-health promotion activities was quoted as total CVD score and total of dyslipidemia practice was quoted as total dyslipidemia score. The CVD and dyslipidemia scores were added to generate a total score for each respondent, respectively. The scores were then further analysed for normality. Both Kolmogorov-Smirnov and Shapiro-Wilk test yield p-value less than 0.05, which indicate that the total CVD and dyslipidemia scores were not normally distributed. Non-parametric tests consisting of Mann-Whitney U test and Kruskal-Wallis test were conducted to determine the significance differences from the demographic characteristics such as gender, mode of practice, ethnicity, location of pharmacy, years of practice, total number of staffs available, completion of dyslipidemia training, background of family members and finally interest in having dyslipidemia training. Meanwhile, for age, Spearman correlation was used. P-value less than 0.05 was considered significance. Significance values (p-value <0.05) showing differences between groups were also extracted. No replacements were made for the missing data. For perceived barriers and contents of educational materials, data were analyzed descriptively using percentage and frequency.

## Results

A total of 312 CPs were involved in this study. This accounts for 91% from the targeted sample size. Table 1 summarizes the demographic data. Majority of the respondents were females (66%), with a mean age (SD) of 32.9 (8.4) years. Most respondents were found to be Malay (52.6%), followed by Chinese (38.8%), Indian (5.1%) and others. For mode of practice, most of the respondents work on full-time basis (86.2%). In terms of experience, the highest percentage was contributed by those with less than 5 years of working experience (62.5%), followed by more than 5–10 years of experience (19.6%). Most of the respondents listed their premises to be located at urban areas (50.6%). The two highest number of respondents were from the West region, Selangor, W.P Kuala Lumpur & Putrajaya (40.7%). This is tailored with the high number of community pharmacies available at the West region. Almost two-third of the respondents claimed to have immediate family members with CVD (63.5%).

**Table 1:** Demographic characteristics of respondents.

Demographic characteristics	Subcategory	Frequency (n)	Percentage (%)
**Age**	Mean = 32.93 SD = 8.435		
**Gender**	Male	106	34
	Female	206	66
**Mode of practice**	Full-time	269	86.2
	Part-time	43	13.8
**Ethnicity**	Malay	164	52.6
	Indian	16	5.1
	Chinese	121	38.8
	Others	8	2.6
**Years of practice**	<5 years	195	62.5
	5–10 years	61	19.6
	11–20 years	29	9.2
	>20 years	27	8.7
**Location of pharmacy**	Rural	40	12.8
	Suburban	114	36.5
	Urban	158	50.6
**Region (state)**	North (Perlis, Kedah, Perak, Penang)	42	18.8
	West (Selangor, WP. Kuala Lumpur & Putrajaya)	127	40.7
	South (Negeri Sembilan, Malacca, Johore)	55	17.6
	East (Kelantan, Terengganu, Pahang)	58	18.5
	Borneo (Sabah, Sarawak)	30	9.6
**Total no. of staffs in the pharmacy**	0	29	9.3
	1–3	184	59.0
	4–5	71	22.8
	>5	27	8.7
	Not valid	1	0.3
**No. of pharmacist in one shift**	1	235	75.3
	More than 1	77	24.7
**Pharmacist completed dyslipidemia training**	Yes	52	16.7
	No	260	83.3
**Interest to join dyslipidemia training**	Yes	279	89.4
	No	7	2.2
	Maybe	26	8.3
**Immediate family members with CVD**	Yes	198	63.5
	No	114	36.5
**Types of cholesterol screening available**	TC only	93	29.8
	TG only	1	0.3
	TC and TG only	5	1.6
	TC, TG, LDL and HDL	175	56.1
	Others	6	1.8
	None of the screening available	32	10.3

In terms of manpower or staffing, most of the respondents had one to three number of staffs at the pharmacy (59%), and most of them had one CP at one shift (75.3%). Regarding dyslipidemia training, majority of the respondents had never attended any dyslipidemia training (83.3%). Regarding the interest to join dyslipidemia training, majority of them expressed their interest for the training (89.4%). For the type of cholesterol tests being offered at the premise, more than half of the respondents (56.1%) provided full cholesterol tests comprising of total cholesterol (TC), triglycerides (TG), low-density lipoprotein (LDL) and high-density lipoprotein (HDL). Meanwhile, 32 of the respondents (10.3%) did not provide any cholesterol test at their premise.

For current involvement in cardiovascular diseases-related health promotion activities and dyslipidemia care services, the results are tabulated in Tables 2 and 3 respectively. The questions were further classified into three main categories which are patient counselling, risk assessment and collaborative care. Among the pertinent information, more than half of the respondents indicated that they never invite other healthcare professionals to screen patients for CVD risk factors (52.9%). In addition, 50.6% of them never invited other healthcare professionals to provide patients with advice or counselling regarding importance of adopting and maintaining healthy lifestyles to prevent CVD. Apart from that, more than half of the respondents (55.5%) mentioned they never provide patients with educational materials on CVD prevention. Looking at cardiac risk assessment tools, 84.3% of them opted for never and rarely in giving patients cardiac risk assessment tools to self-assess their cardiac risk. This finding is tailored with another statement in the similar section where 73.7% chose never or rarely in assessing patients’ individual risk for cardiovascular diseases using cardiac risk assessment tools. Other elements of health promotion activities showed satisfactory levels of practice where more than 50% of the respondents opted for often and always.

**Table 2:** Current involvement in cardiovascular diseases-related health promotion activities.

QUESTION. On average how much do you involve in the following activities:	NEVER n(%)	RARELY n(%)	OFTEN n(%)	ALWAYS n(%)
**Patient Counselling**				
Respond to patient inquiries relating to cardiovascular diseases prevention including hypertension and dyslipidemia.	1(0.3)	42(13.5)	188(60.3)	81(26)
Provide patients with advice or counselling regarding the importance of adopting and maintaining healthy lifestyles to prevent cardiovascular diseases	5(1.6)	30(9.6)	175(56.1)	102(32.7)
Provide patients with advice or counselling on the importance of screening and early detection of cardiovascular diseases’ risk factors including hypertension and dyslipidemia.	4(1.3)	42(13.5)	175(56)	91(29.2)
Provide patients with educational materials about cardiovascular diseases prevention including hypertension and dyslipidemia management (educational materials may include brochures, flyers, pamphlets, posters and buttons).	37(11.9)	136(43.6)	103(33)	36(11.5)
**Risk Assessment**				
Give patients cardiac risk assessment tools to self-assess their cardiac risk	123(39.4)	140(44.9)	36(11.5)	13(4.2)
Assess patients’ individual risk for cardiovascular diseases using cardiac risk assessment tools.	117(37.5)	113(36.2)	63(20.2)	19(6.1)
Screen patients for the presence of cardiovascular diseases’ risk factors including hypertension and dyslipidemia in the pharmacy	33(10.6)	87(27.9)	145(46.5)	47(15.1)
**Collaborative Care**				
Invite other health care professionals (e.g. nurse, dietitian, doctor…) to screen patients for cardiovascular diseases’ risk factors including hypertension and dyslipidemia in pharmacy.	165(52.9)	99(31.7)	41(13.1)	7(2.2)
Invite other health care professionals (e.g. nurse, dietitian, doctor…) to provide patients with advice or counselling regarding the importance of adopting and maintaining healthy lifestyles to prevent cardiovascular diseases	158(50.6)	106(34)	39(12.5)	9(2.9)
Refer patients to doctors for cardiovascular diseases’ management including hypertension and dyslipidemia when needed	37(11.9)	58(18.6)	143(45.8)	74(23.7)

**Table 3:** Current involvement of CP in dyslipidemia care services.

QUESTION On average, how much are you involve in the following activities:	NEVER n(%)	RARELY n(%)	OFTEN n(%)	ALWAYS n(%)
**Patient Counselling**				
Counsel about the cautions of over-the-counter drugs or herbal products as they relate to dyslipidemia management	18(5.8)	47(15.1)	162(51.9)	85(27.2)
Emphasize on the importance of timely measurement of blood lipids/cholesterols	7(2.2)	52(16.7)	160(51.3)	93(29.8)
Counsel the patient about the desired therapy outcomes	8(2.6)	64(20.5)	152(48.7)	88(28.2)
Discuss the potential benefits of anti-dyslipidemic drugs	11(3.5)	55(17.6)	167(53.5)	79(25.3)
Describe the appropriate time to administer each anti-dyslipidemic drug	7(2.2)	37(11.9)	152(48.7)	116(37.2)
Describe common adverse effects of each anti-dyslipidemic drug	13(4.2)	59(18.9)	153(49)	87(27.9)
Provide education on the importance of regular screening for heart disease	12(3.8)	73(23.4)	149(47.8)	78(25)
Stress the importance of lifestyle modifications (e.g. diet, exercise, weight management where applicable, etc…) in dyslipidemia management	3(1)	29(9.3)	143(45.8)	137(43.9)
Promote smoking cessation where applicable	21(6.7)	70(22.4)	138(44.2)	83(26.6)
Counsel on current recommendations for antiplatelet therapy	54(17.3)	129(41.3)	96(30.8)	33(10.6)
**Risk assessment**				
Evaluate blood lipid log for values outside target range	45(14.4)	102(32.7)	106(34)	59(18.9)
**Collaborative Care**				
Counsel on when to contact the health care provider regarding cholesterol control	15(4.8)	70(22.4)	154(49.4)	73(23.4)
Refer dyslipidemia patients to a dietitian	114(36.5)	118(37.8)	61(19.6)	19(6.1)
Provide drug therapy recommendations to the doctor to help the patient reach blood lipid targets	111(35.6)	115(36.9)	65(20.8)	20(6.7)
**Adherence assessment and recommendation**				
Review the patient’s drug refill history to evaluate adherence to drug therapy	44(14.1)	97(31.1)	114(36.5)	57(18.3)
Provide patient-specific interventions to help improve adherence to drug therapy	22(7.1)	104(33.3)	135(43.3)	51(16.3)

In the next section, respondents were asked about their provision of dyslipidemia care services. The findings are tabulated in Table 3. Some of the statements indicated a lack of practice. One example is collaborative care, where 72.5% never or rarely provided drug therapy recommendations to the doctor in helping patients to reach blood lipid targets. This is also tailored with another statement where 74.3% of respondents mentioned they never or rarely referred patients with dyslipidemia to dietitians. Another finding revealed that almost half of the respondents (47.1%) never or rarely evaluated blood lipid logs for values outside the target range. In terms of checking patients’ medical history, 45.2% of respondents opted for never or rarely reviewing the patients’ drug refill history to evaluate adherence to drug therapy.

As described in Table 4, few sociodemographic parameters were seen to be significant. For ethnicity, Chinese respondents show significantly higher practice (both CVD and dyslipidemia) compared to the other races. For the location of pharmacy, community pharmacists working in urban areas show the highest practice, followed by rural areas and suburban areas with the least practice. Pharmacies with more than one pharmacist available in one shift show significantly higher practice compared to only one pharmacist available. In terms of completion of dyslipidemia training, CVD and dyslipidemia practice were significantly higher among those who had completed the training. Other demographic characteristics showed no significant differences between groups.

**Table 4:** Significance differences of demographic characteristics with total CVD/dyslipidemia score.

Independent variable	Subcategory	Total CVD score, mean (SD)	p-value	Total dyslipidemia score	p-value
**Age**	-	14.42(5.21)	0.498^#^	26.65(8.70)	0.249^#^
**Gender**	Male	14.95(5.28)	0.364^†^	26.58(8.17)	0.685^†^
	Female	14.18(5.17)		26.7(8.96)	
**Mode of practice**	Full-time	14.64(5.29)	0.126^†^	26.87(8.67)	0.373^†^
	Part-time	13.23(4.59)		25.35(8.79)	
**Ethnicity**	Malay	13.26(4.82)	<0.001^‡^*	24.80(8.31)	<0.001^‡^*
	Indian	15.38(6.37))		32.00(10.69)	
	Chinese	16.05(5.22)		28.87(8.24)	
	Others	12.88(5.44)		21.50(8.07)	
**Years of practice**	<5 years	14.06(5.21)	0.253^‡^	26.10(9.06)	0.397^‡^
	5–10 years	15.08(5.13)		26.79(7.52)	
	11–15 years	17.25(6.33)		29.08(7.09)	
	16–20 years	14.82(5.75)		29.24(7.93)	
	>20 years	14.30(4.38)		27.67(9.41)	
**Location of premise**	Rural	14.55(6.0)	0.025^‡^*	26.58(9.15)	0.002^‡^*
	Suburban	13.39(4.58)		24.46(8.25)	
	Urban	15.17(5.34)		28.27(8.57)	
**State**	North (Perlis, Kedah, Perak, Penang)	14.60(4.95)	0.710^‡^	27.63(8.74)	0.450^‡^
	West (Selangor, W.P Kuala Lumpur & Putrajaya)	14.71(5.33)		27.12(7.88)	
	South (Negeri Sembilan, Malacca, Johore)	15.40(5.82)		27.91(9.66)	
	East (Kelantan, Terengganu, Pahang)	13.38(4.83)		24.55(8.97)	
	Borneo (Sabah, Sarawak, W.P Labuan)	13.40(4.36)		25.10(9.06)	
**Total no. of pharmacy assistants**	0	14.10(4.48)	0.468^‡^	26.45(6.63)	0.530^‡^
	1–3	14.42(5.26)		26.51(8.98)	
	4–5	14.07(4.79)		26.15(8.57)	
	>5	15.56(6.41)		28.93(8.95)	
**No. of pharmacist in one shift**	1	13.75(4.68)	<0.001^†^*	26.00(8.39)	0.012^†^*
	More than 1	16.58(6.17)		28.70(9.29)	
**Completion of dyslipidemia training**	Yes	16.52(5.77)	0.002^†^*	30.73(7.77)	<0.001^†^*
	No	14.03(5.00)		25.84(8.64)	
**Interest to join dyslipidemia training**	Yes	14.46(5.31)	0.957^‡^	26.53(8.84)	0.656^‡^
	No	15.00(6.76)		25.29(8.30)	
	Maybe	14.12(3.63)		28.35(6.99)	
**Immediate family members with CVD**	Yes	14.48(5.23)	0.628^†^	27.27(8.66)	0.104^†^
	No	14.37(5.21)		25.60(8.67)	

*Statistically significant difference, p<0.05

^#^Spearman’s rho correlation test

^†^Mann-Whitney U test

^‡^Kruskal-Wallis test

Looking at the perceived barriers as described in Table 5, the highest percentage was contributed by lack of access to medical records (71.2%), followed by lack of CVD-related educational materials, with 70.8% of respondents indicating this. Low patient expectations regarding pharmacists’ role in CVD prevention is contributed by 43.9% of the CPs. Meanwhile, the least percentage of perceived barriers is contributed by no interest in providing CVD prevention services with only 1.9% and culture and religious barriers with 4.8%.

**Table 5:** Perceived barriers in providing cardiovascular diseases prevention services.

PERCEIVED BARRIERS TO PROVIDING CARDIOVASCULAR DISEASES PREVENTION SERVICES	Percentage (%)(n)
Lack of access to medical records	71.2(n = 222)
Lack of CVD-related educational materials such as flyers, brochures, etc.	70.8(n = 220)
Low patient expectations regarding pharmacist’s role in CVD prevention	43.9(n = 137)
Lack of communication with other healthcare providers	42.6(n = 133)
Lack of time	40.1(n = 125)
Lack of CVD therapeutic knowledge and/or skills	34.9(n = 109)
Shortage of personnel	32.8(n = 102)
Lack of management and government support	31.7(n = 99)
Limited funding	23.4(n = 73)
Lack of patients with CVD risk factors who visit the pharmacy	21.2(n = 66)
Lack of a private counselling area	17.9(n = 56)
Cultural and religious barriers	4.8(n = 15)
Lack of interest in providing CVD prevention services	1.9(n = 6)

In the final section, the CPs were asked about the contents of educational materials in dyslipidemia management that they wanted to know more about. They may choose more than one answer. As shown in Table 6, the highest content was contributed by the risk assessment tools for CVD, and the least content of interest was contributed by the recommendation for referral to other healthcare professionals.

**Table 6:** Interest of CPs in terms of contents of educational materials.

CONTENTS OF EDUCATIONAL MATERIALS	Percentage (%) (n)
**Risk assessment tools for CVD**	86.5(n = 270)
**Monitoring in terms of efficacy and side effects (lipid-lowering therapy/supplements/target lipid level)**	79.1(n = 246)
**Optimization of therapeutic lifestyle changes (TLC) in dyslipidemia (eg. dietary intake, exercise and smoking cessation).**	74.4(n = 232)
**Screening for dyslipidemia**	73.1(n = 228)
**Adherence assessment (those with lipid-lowering therapy/supplement intake)**	70.2(n = 219)
**Pharmacological management of dyslipidemia**	69.9(n = 218)
**Recommendation for referral to other healthcare professionals (physicians/dietitians)**	68.6(n = 214)

## Discussion

Based on the findings, it can be suggested that CPs provide satisfactory practice in some elements; however, a lack of practice was seen in a few areas. This could be due to several reasons which may contribute to the current practice of CPs in Malaysia. Strengths and barriers encountered by CPs were further discussed in the section below.

### Patient counselling

For CVD-related health promotion activities, our study reported that CPs exhibited satisfactory practice in providing counselling. However, this provision of counselling was done without the usage of any educational materials. This is consistent to barriers perceived by CPs in which a lack of CVD-related educational materials was reported as the second highest barrier. From this study, it can be assumed that the CPs are using their own materials or did not use any educational materials to ensure the effectiveness of the counselling. This finding then serves as an opportunity to explore the comprehension of patients to the counselling provided by the CPs. When patients can comprehend and grasp the information, it will enable them to improve their adherence towards the recommendations provided. The opportunity for educational materials is discussed further in the perceived barriers section below.

### Risk assessment

For CVD-related promotional activities, the majority of CPs did not use any cardiac risk assessment tool or provide patients with the risk assessment tool to be explored by themselves. Similarly, in dyslipidemia care services, most of the CPs were reported to never or rarely analyse the lipid profile once it is outside the targeted range. Nevertheless, majority of CPs reported being frequently involved in screening patients’ CVD risk factors such as hypertension and dyslipidemia. This might be due to the availability of a fully automated sphygmomanometer and a point-of-care cholesterol testing device on the premise. This practice is aligned with World Health Organization which recommends early detection and management of people who are at high risk of developing CVD [20]. To measure and control cardiovascular risk, it is necessary to establish and advocate highly accessible screening programmes implemented in the community settings.

Upon providing recommendations for prevention and therapy, it is recommended to have a precise estimate of the absolute risk of experiencing a first CVD incident using instruments like the Framingham Risk Score [21]. Although the Framingham Risk Score is available in the national guideline for CVD and dyslipidemia, the practice of using the risk assessment tool is still found to be lacking. This might be due to the fact that CPs in Malaysia did not receive appropriate training to perform the risk assessment. Improved knowledge and skills are expected after the provision of training or any educational programme. This is supported by a study conducted in 2020 by Zolezzi and colleagues, who conducted a workshop series to educate CPs about CVD risk assessment measures. At the end of their study, the authors concluded that the educational programme had successfully improved CPs’ knowledge and skills related to CVD [21].

### Collaborative care

For the perceived barriers, almost half of the CPs agreed that there is a lack of communication with other healthcare providers. As communication is an integral part of collaborative care, this can be interpreted as current interdisciplinary teamwork among healthcare professionals in the private sector is still weak. This might be due to several challenges encountered by healthcare professionals to work collaboratively. The collaboration of healthcare experts in diverse locations, such as private clinics and community pharmacies, can be challenging. However, this situation is different in the hospital setting, where efficient communication has been observed to happen regularly in Malaysia. This could be because all departments are contained within one healthcare institution by nature, and all patient information is computerized and centralized.

Collaborative care in treating patients with cardiovascular disease offers a multifaceted approach that significantly offers benefit and enhances patient outcomes. A recent systematic review revealed a successful impact of such intervention on patients’ psychosocial or cognitive component of mental health status. Along with the cholesterol levels, the behavioural outcomes of caloric intake and physical activity also significantly improved in studies practising collaborative care approach [22].

Collaborative care landscape is still evolving to address the healthcare needs of patients with dyslipidemia. Unfortunately, a study conducted by Mubarak et al. (2019), asserted that general practitioners and CPs collaborate the least in Malaysia [23]. To navigate this issue, the readiness of healthcare professionals should be examined further. A study was conducted by Roslan et al., regarding the perception from medical doctors about collaborative care [24]. The participants of the study agreed that collaboration is key to ensuring both the best possible health outcomes for patients and the well-being of medical personnel [30]. Several participants also suggested that medical doctors should exhibit attributes of strong leadership, good communication and outstanding behaviours when working together with other health professionals [24].

### Perceived barriers to providing cardiovascular diseases prevention services

#### Lack of access to medical records and lack of communication with healthcare providers

In Malaysia, as there is no centralized computerized system that links patient information to community pharmacies which poses a challenge for the CPs to access patients’ medical records. Nevertheless, it can be a good practice for CPs to encourage patients to bring their medications, appointment books, referral letters, medical reports or any necessary relevant documents. This could facilitate the counselling process and provide CPs with a thorough understanding of the patient’s condition.

Looking at the practice in other countries, patient information can be easily accessible in a computerized and centralized system. The use of computerised drug administration data can be used to prevent pharmaceutical dispensing errors [25]. Patients’ medical records can be accessed online, and prescribers could be contacted for further information about the patient’s condition and prescription. This synchronization and integration mechanism will lead to better therapeutic management provided to patients. System integration and compliance are important components towards the achievement of safe medication use [25]. From here, it was also possible to solve the barrier of lack of communication with other healthcare providers as 42.6% of CPs agreed about this statement.

In addition, another effort that could be proposed is the implementation of dispensing separation. In fact, for an efficient system of checks and balances, diagnosis and prescription are two complex jobs that should be performed by two different healthcare experts [26]. This eliminates the tendency to favour particular products or to overprescribe when there is financial temptation, thereby encouraging evidence-based and rational usage of drugs [26]. A study conducted in Malaysia highlighted that, a collaborative paradigm of chronic care should be gradually adopted by doctors. In Malaysia, controlling chronic diseases should involve collaboration between CPs and general practitioners or doctors [26]. In the similar study, the authors made recommendations for the CPs whereby they should increase their clinical expertise and awareness of particular chronic diseases. As a result, before beginning the service, CP must obtain a valid accreditation for training, a certification, or a course for a particular chronic disease such as hypertension, diabetes mellitus or asthma [23].

#### Availability of educational materials

The majority of CPs agreed that the lack of educational materials is one of the barriers encountered by them. Exploring the type of educational materials that should be provided, Lee and colleagues (2021), in their asthma management study in Malaysia, also highlighted the necessity of educational materials. Participants in the study who are medical doctors suggested for a visual format of asthma action plans rather than in excessively wordy manner [27]. The healthcare professionals also identified the dislike towards reading as a typical Asian cultural trait. Some people believed that patients would understand asthma action plans better if they were presented in a more visual style (such as images or animation) [27]. According to previous research, graphical format tools have been demonstrated to improve consultations and make it easier for people to grasp management plans, including in Malaysia [28]. Therefore, the preparation of graphical and visual educational materials shall be provided with patient-centred approaches for the CPs in the future.

#### Low patient expectations regarding pharmacist’s role in CVD prevention

A systematic review by El Hajj et al. 2019 revealed that in Qatar, buying over-the-counter or obtaining prescription medications are the main reasons people go to community pharmacies [29]. Another study conducted in Kuwait in 2017, reported that most people come to the pharmacy to purchase either prescription or non-prescription medications only. Majority of the public did not expect that the pharmacist’s responsibility would involve monitoring patients’ health status and ensuring the safe and appropriate use of medications [30].

A more recent local study in East Malaysia reported that out of 647 respondents, more than half of the respondents (54.4%) believed that a pharmacist’s only responsibility was to dispense prescriptions. A total of 66.6% of the respondents believed that a pharmacist’s job was to follow doctors’ instructions [31]. Nevertheless, the majority of the respondents believed that pharmacists may play a bigger role in healthcare in the future, and 89.0% of respondents appreciated and followed the advice given by their pharmacists [32]. From these findings, it can be highlighted that the advanced role of CPs in providing CVD and dyslipidemia services is yet to be known by the public. More efforts, specifically on advertising awareness campaigns, should be done to promote such services to the nearby community.

#### Lack of time

Lack of time was the fifth barrier encountered by CPs. Excessive workload may be one of the factors that contributed to this. A review done in the United Kingdom reported that pharmacists spend most of their time dispensing medicines [33]. Apart from that, Alkhateeb and his colleagues in West Virginia found that pharmacists’ capacity to spend time with patients was being hampered by their growing workload [34]. Exploring this, an observational study which analysed the workload among Portuguese CPs was conducted in 2016 [34]. According to their study, most of the pharmacists’ time (32.7%) was spent on administrative tasks (e.g. inventory and stock control),. with limited time was spent on dispensing medications and screening services such as blood pressure monitoring, uric acid and cholesterol tests [35]. The authors from the same study also asserted that CPs should delegate administrative tasks and routine chores to other pharmacy staff as a high percentage of time was spent on these activities.

#### Lack of therapeutic knowledge and skills

It is found that the majority of CPs never completed any dyslipidemia training previously. Provision of training is crucial, which is also supported by the data where completion of dyslipidemia training shows significant differences in terms of CVD and dyslipidemia practice. In order to enhance pharmacists’ lifelong learning, the Malaysian Academy of Pharmacy offers opportunities for ongoing continuing professional development (CPD) to working pharmacists [36]. This CPD platform should be filled with more CVD and dyslipidemia training or workshops. CPs who completed these training sessions could be certified to perform CVD and dyslipidemia practices. This is to ensure that the best management and recommendations could be provided to the patients with the ultimate goal to increase patients’ trust in CPs and improve treatment outcomes.

#### Shortage of personnel (number of community pharmacists and pharmacy assistants)

Shortage of personnel is one of the important barriers encountered in our findings. It is shown that the total number of pharmacy assistants does not provide a significant difference in terms of CVD and dyslipidemia practice scores. However, the number of pharmacists available on the premise make a significant difference towards the practice. This shows that the pharmacy assistants’ role may not be extended in CVD and dyslipidemia practices. According to the Community Pharmacy Benchmarking Guideline issued by the Ministry of Health in 2016 [37], the preparation of medications for dispensing is under the responsibility of the pharmacy assistant. However, scheduled poisons cannot be dispensed without a pharmacist present [37]. This might explain why pharmacy assistants do not perform the CVD-related health promotion and dyslipidemia care services in regular basis. Thus, the high total number of pharmacy assistants did not lead to higher provision of CVD-related health promotion and dyslipidemia care services in our setting. More administrative tasks such as inventory control, marketing and merchandising activities could be managed and handled by pharmacy assistants instead. This will allow more time and flexibility for CPs to focus on disease prevention measures such as health screening and the provision of medication counselling. At the same time, community pharmacies’ owners must identify the number of personnel appropriate for their premises. A sufficient number of personnel will enable services related to disease prevention could be delivered more effectively to the public.

### Limitations

There are several limitations in this study. Firstly, the data collected was based on self-reports by CPs which may lead to social desirability bias. Secondly, the number of respondents from each region is not similar due to the heterogenicity of the number of community pharmacies available. West region comprising W.P Kuala Lumpur, Selangor and W.P Putrajaya have more community pharmacies in comparison to other regions. Nevertheless, this study managed to include sufficient representatives from all the 14 states in Malaysia. To the best of our knowledge, this is the first study conducted in Malaysia to determine the practice and perceived barriers faced by CPs in the provision of CVD-related promotional activity and dyslipidemia services. Future qualitative studies are highly recommended to explore any other issues or problems that occur in the setting. This will also enable the problems identified such as lack of manpower, lack of training and educational materials or other possible problems to be solved from the root causes.

## Conclusion

Community pharmacists (CPs) in Malaysia play a valuable role in facilitating CVD-related health promotion activities and providing dyslipidemia care services, particularly in patient counseling. However, the scope of their contributions should be expanded. Several areas have shown a need for improvement, including risk assessment, the potential for collaborative care, access to medical records, knowledge and skills, the availability of educational materials, time constraints, and a shortage of personnel, all of which pose significant barriers. Enhancing CP practices could be achieved through the implementation of a centralized and computerized system within the national private sector. Additionally, providing ample educational resources, refining marketing strategies, delegating certain administrative responsibilities to pharmacy assistants, and offering comprehensive training on CVD and dyslipidemia management may mitigate these challenges and elevate CP practice standards. These strategic measures are expected to further empower CPs and contribute to our overarching goal of reducing overall CVD-related mortality and morbidity.

## Supporting information

S1 Data (XLSX)
